# Minimum Information About a Microarray Experiment (MIAME) – Successes, Failures, Challenges

**DOI:** 10.1100/tsw.2009.57

**Published:** 2009-05-29

**Authors:** Alvis Brazma

**Affiliations:** Microarray Informatics Group, European Bioinformatics Institute, EMBl-EBI, Wellcome Trust Genome Campus, Hinxton, Cambridge, UK

**Keywords:** microarrays, next-generation sequencing, bioinformatics, biomedical data management, data availability, experiment reproducibility

## Abstract

The Minimum Information About a Microarray Experiment (known as MIAME) guidelines describe information that needs to be provided to enable the interpretation of the results of a microarray-based experiment unambiguously. The MIAME guidelines were developed by the Microarray Gene Expression Data (MGED) Society. Since the MIAME position paper was published in 2001, it has been cited in the scientific literature well over a thousand times. MIAME has been replicated for many other technologies, the major data repositories are supporting MIAME, and most scientific journals have adopted MIAME guidelines as a requirement for publishing. With the advent of new-generation sequencing technology, MIAME faces new challenges. To address this, the MGED Society has proposed new guidelines, i.e., Minimum Information about a high-throughput SeQuencing Experiment (MINSEQE). Here we present analysis of the reasons for the success of MIAME, as well as discuss where it has failed, and the challenges it faces.

## INTRODUCTION

It has been 8 years since the paper "Minimum Information About a Microarray Experiment (MIAME) – Toward Standards for Microarray Data" was published[1]. It stated the obvious – if conclusions based on microarray data analysis are to be verified, then not just the supporting raw data should be made available, but also it should be revealed what nucleotide sequences were present on the array, what the assayed samples were and how they were treated, which sample was processed on which array and which data file was obtained in the result, and how were the data processed. So why does the MIAME publication have over a 1000 citations?

Microarrays were the first high-throughput technology used to assay and compare different biological conditions, different organs or cell types, and different individuals. Data from such assays had value only if the biological properties of the samples and phenotypes that were assayed were recorded along side the data obtained by these assays. The Human Genome Project and DNA sequencing projects, which by the standards of these days were already producing large amounts of data, did not have this problem – the reference genome was the same for the organism regardless of organism's physiological condition. For microarrays, the biological state of the system, the sample treatment, and even the experimental procedures mattered. Moreover, the study designs were often quite complex, and given the amounts of data produced in a single experiment, it was not sufficient to capture this information just somehow. To make the data usable in analysis, everything had to be recorded systematically. MIAME stated this explicitly. It marked the start of the high-throughput biology and a change of the paradigm – it was no longer only the laboratory work that was nontrivial, but also the management and processing of the data.

## SUCCESSES

However, what made MIAME important was that it went on a successful mission, i.e., in the age of high-throughput biology, to ensure the continuation of the well-established principle in science that data supporting published conclusions must be made available *in a way that makes these data usable for others*. The MGED Society (www.mged.org) published a letter[2] calling for mandatory submission of MIAME-compliant data to the public repositories ArrayExpress[3], CIBEX[4], or Gene Expression Omnibus (GEO)[5]. A simple MIAME checklist was created (www.mged.org/miame) and most of the major scientific journals adopted this principle. By now, data from over 10,000 different microarray studies have been deposited into these public repositories. The repositories, in turn, are supporting the archiving of MIAME-compliant data. These data are used by other researchers not only to compare and combine them with their own data, or to test new methods, but also to build secondary "value added" databases, such as GENEVESTIGATOR[6], ONCOMINE[7], or the ArrayExpress Gene Expression Atlas[8]. These databases curate, analyse, transform, and make microarray data accessible to every biologist everywhere in the world regardless of whether they are experts in microarray data analysis or not. These resources provide an interface as simple as Google to make queries such as: "where is my favourite gene expressed" or "the expression of which genes is changed in a disease I am interested in".

The MIAME initiative has prompted the creation of similar guidelines for the whole range of high-throughput technologies, e.g., MIAPE (Minimum Information About a Proteomics Experiments), MISFISHIE (Minimum Information Specification For In Situ Hybridization and Immunohistochemistry Experiments), or particular communities, e.g., MIAME-tox (MIAME for toxicology). A special portal called MIBBI (Minimum Information about Biological and Biomedical Investigations[9]) has been established to assist such initiatives. MIAME and its "derivatives" in MIBBI have not only facilitated data sharing, but they have guided software development, making it easier to design databases to capture all the necessary data and metadata for various high-throughput technologies.

## FAILINGS

Several studies have recently shown (e.g., [10,11]) that despite the wide adoption of MIAME principles by scientific journals, it has often been difficult to get hold of microarray data on which the publications were based. Even when data were available, it has not always been possible to reproduce the results, mostly because the data were not MIAME compliant[11]. Implementing MIAME requirements has turned out to be challenging. The situation could be helped if journals required that data should be submitted to public repositories and the respective repository accession numbers be provided at the time when the manuscript itself is submitted for review. This would allow the reviewers to inspect the data anonymously and to benefit from the services offered by the repositories (e.g., [12]).

MIAME is only a set of guidelines of what information needs to be captured. MIAME does not provide, nor is intended to provide, a format for representing this information and data. Without a standard computer-readable format, the utility of these data is limited; however, no MIAME-supportive format is as widely adopted as MIAME itself. Soon after MIAME was published, a standard XML-based format (MAGE-ML) was proposed[13], but it did not gain popularity, largely due to it its high complexity. Specials tools were needed to read and write it, and the development of such tools turned out to be too expensive and slow to come. More recently, a much simpler MIAME-supportive format, called MAGE-TAB[14], has gained popularity. It is easy to view and edit MAGE-TAB documents without special tools, it is used by ArrayExpress, and it can be imported in data analysis tools like Bioconductor. A more general format, ISA-TAB[15], has been proposed for multiomics studies. One of the lessons we learned is that developing and adopting computer-readable standards is more difficult than adopting general guidelines, and that the simplicity is the key to success[16].

## CHALLENGES

Microarrays were arguably the first high-throughput technology for biological and biomedical research. Many others such technologies have been developed recently; most importantly, the new-generation sequencing (NGS). With the costs of NGS decreasing, it has the potential to take over many of the microarray applications, including gene expression profiling and epigenetic and gene copy number variation assays. The MGED Society has already proposed draft guidelines for MINSEQE (http://www.mged.org/minseqe/). ArrayExpress and GEO are accepting NGS-based data and are working towards finalising the details of MINSEQE implementation.

Adopting MINSEQE may be even more challenging than MIAME. First, sequencing is used in many different ways, not only to assay biological sates, but also, for instance, to resequence genomes or metagenomes. Therefore, MINSEQE may not be relevant for every experiment. The journals may find it difficult to decide in which cases MINSEQE-compliant data submissions to ArrayExpress or GEO should be required. Second, when sequencing human DNA or RNA, there may be legitimate data privacy issues – long-enough sequences may make the individual identifiable. In such cases, only deidentified processed data may be made public, rather than the raw sequences. However, despite these difficulties, the scientific journals should adopt and support MINSEQE without delay. It should be required that data from NGS experiments that assay or compare different biological states or conditions, e.g., gene expression (so-called RNAseq) assays, should be submitted to ArrayExpress or GEO prior to publication. If MINSEQE adoption does not happen soon, there is a danger of losing the MIAME achievements, which has enabled every biologist to benefit from functional genomics data generated by others.
